# Highlighting a histopathological dilemma: atypical dermatofibrosarcoma protuberans diagnosed with multimodal dermoscopy

**DOI:** 10.1093/skinhd/vzaf113

**Published:** 2026-01-28

**Authors:** Varun H, Adarshlata Singh, Ambika Kondalkar, Bhushan Madke, Vangala Naga Nitya, Anurag Mittal, Gayathri Bomma Reddy

**Affiliations:** Department of Dermatology, Venereology and Leprosy, Datta Meghe Institute of Higher Education and Research, Jawaharlal Nehru Medical College, Wardha, Maharashtra, India; Department of Dermatology, Venereology and Leprosy, Datta Meghe Institute of Higher Education and Research, Jawaharlal Nehru Medical College, Wardha, Maharashtra, India; Department of Dermatology, Venereology and Leprosy, Datta Meghe Institute of Higher Education and Research, Jawaharlal Nehru Medical College, Wardha, Maharashtra, India; Department of Dermatology, Venereology and Leprosy, Datta Meghe Institute of Higher Education and Research, Jawaharlal Nehru Medical College, Wardha, Maharashtra, India; Department of Dermatology, Venereology and Leprosy, Datta Meghe Institute of Higher Education and Research, Jawaharlal Nehru Medical College, Wardha, Maharashtra, India; Department of Dermatology, Venereology and Leprosy, Datta Meghe Institute of Higher Education and Research, Jawaharlal Nehru Medical College, Wardha, Maharashtra, India; Department of Dermatology, Venereology and Leprosy, Datta Meghe Institute of Higher Education and Research, Jawaharlal Nehru Medical College, Wardha, Maharashtra, India

## Abstract

An adult male agricultural worker presented with an asymptomatic, heterogeneously pigmented, indurated nodule featuring multiple protuberances on the left pelvic skin. Cross-polarized dermoscopy revealed pink structureless areas and a hyperpigmented reticular network; yellow light dermoscopy accentuated the pigmentary network and entangling vasculature. Ultraviolet-induced fluorescence dermoscopy demonstrated blue–white autofluorescence of surface keratin and milky-white fluorescence of the collagenous/myxoid stroma. Histopathology showed a monomorphic spindle-cell infiltrate without atypia; immunohistochemistry (IHC) confirmed a CD34-positive, S100-negative spindle-cell neoplasm consistent with a myxoid variant of dermatofibrosarcoma protuberans. This case highlights the diagnostic utility of multimodal dermoscopy integrated with histopathology and supportive IHC in precisely evaluating atypical cutaneous tumours.

## Teaser

An adult with a protuberant cutaneous nodule underwent multimodal dermoscopy, revealing features suggestive of dermatofibrosarcoma protuberans (DFSP). Despite inconclusive histopathology, immunohistochemistry confirmed a myxoid variant of DFSP. This case highlights the critical integration of multimodal dermoscopy, histology and advanced diagnostics to resolve challenging cutaneous tumours.

What is already known about this topic?

- Dermatofibrosarcoma protuberans (DFSP) is a rare, locally aggressive sarcoma that can present with variable clinical and histopathological features, which may resemble benign spindle-cell tumours.
- Histopathology, supported by immunohistochemistry (IHC), remains the diagnostic gold standard for DFSP.
- Noninvasive imaging modalities have increasingly been used to enhance diagnostic accuracy and guide biopsy site selection.
- Multiple dermoscopic patterns have been variably described in DFSP, with no pathognomonic feature.

What does this study add?

- This case illustrates how multimodal dermoscopy can complement histopathological assessment, particularly in diagnostically challenging variants of DFSP.
- Ultraviolet-induced fluorescence dermoscopy offered additional insights into the lesion’s stromal composition for targeted sampling.
- Combined clinicopathological and IHC correlation enabled a confident diagnosis, despite overlapping benign features.
- The report highlights dermoscopy’s role in enhancing biopsy planning and diagnostic accuracy in spindle-cell tumours.

Dermatofibrosarcoma protuberans (DFSP) is a rare, locally aggressive cutaneous sarcoma of fibroblastic/myofibroblastic origin with a characteristic *COL1A1–PDGFB* fusion [t(17;22)], accounting for <0.1% of all malignancies and ∼1% of soft-tissue sarcomas.^1^ It typically presents in adults aged 20–50 years as a slow-growing, firm, skin-coloured fleshy papule or nodule on the trunk or proximal extremities, and is classified as an ­intermediate-grade malignancy.

Histologically, DFSP shows a monomorphic spindle-cell infiltrate in a storiform (cartwheel) pattern with finger-like projections into the subcutis, producing the classic ‘­honeycomb’ pattern.^2^ Although metastasis is rare, recurrence occurs in 2–21% of cases, especially those with positive margins.^3^ Myxoid and pigmented variants may mimic benign spindle-cell lesions, underscoring the role of ­noninvasive multimodal dermoscopy as a complementary tool to ­clinicopathological evaluation.^4^

## Case report

A 40-year-old man presented to the dermatology clinic with a 10-month history of a slowly enlarging, asymptomatic lesion on his left pelvic region, initially noted as a brown spot that gradually became raised and developed heterogeneous colouration without discharge, pain or pruritus. Head-to-toe examination and systemic examination did not reveal any significant abnormality.

**Figure 1:**
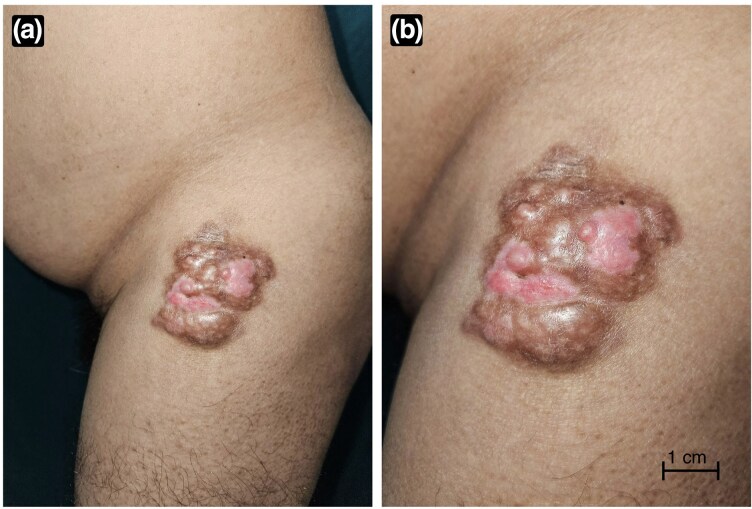
(a) Clinical image depicting an ill-to-well defined heterogeneously pigmented nodular plaque. (b) Close-up view of the same lesion, showing fleshy surface and multiple protuberances.

**Figure 2:**
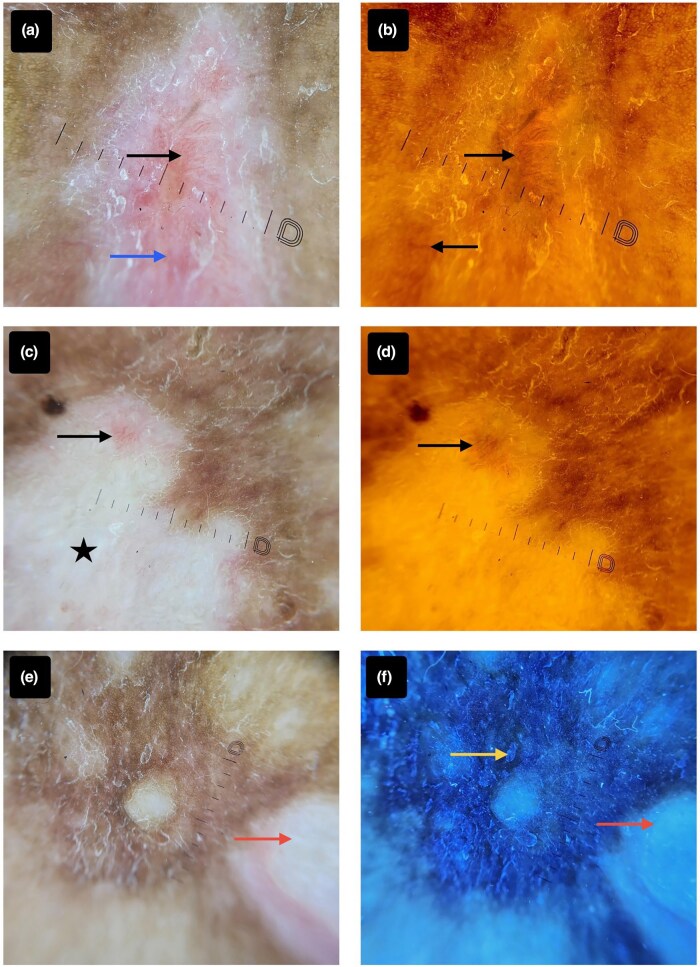
(a) Contact dermoscopy in cross-polarized light (DermLite DL5 coupled with an iPhone 16 camera) reveals multiple arborizing vessels (black arrow) and pink structureless areas (blue arrow). (b) Contact dermoscopy using yellow light (DermLite DL5 580 nm coupled with an iPhone 16 camera) demonstrates accentuated vascular structures (black arrows) and a hyperpigmented peripheral, lacy pigmentary network. (c) Contact dermoscopy under cross-polarized light (DermLite DL5 coupled with an iPhone 16 camera) shows entangled vessels (black arrows) in a white structureless area (black star). (d) Contact dermoscopy with yellow light (DermLite DL5 580 nm coupled with an iPhone 16 camera) highlights the prominence of entangled vessels (black arrows). (e) Contact dermoscopy in cross-polarized light (DermLite DL5 coupled with an iPhone 16 camera) displays multiple white structureless areas (red arrow). (f) Ultraviolet-induced fluorescence dermoscopy (DermLite DL5 Wood mode 365 nm coupled with an iPhone 16 camera) of the same field exhibits yellow–blue fluorescence from keratin’s autofluorescence (yellow arrow) alongside milky-white collagen fluorescence, indicative of a collagenous/myxoid stromal composition. (Scale bar = 1 mm.)

Cutaneous examination revealed an ill-defined, firm, ­nontender, indurated plaque approximately 3–4 cm in diameter with a lobulated, glossy, slightly scaly surface and mixed pigmentation (Figure 1). Cross-polarized dermoscopy (DermLite DL5; DermLite, Aliso Viejo, CA, USA) demonstrated a well-demarcated lesion with a delicate reticular pigmentary network, pink-to-white structureless areas and entangling serpentine-to-arborizing vessels. Yellow light dermoscopy (DermLite DL5 580 nm) further enhanced the contrast of melanin and haemoglobin, highlighting pigmentary network and vascular channels against a yellow–orange background. Ultraviolet-induced fluorescence dermoscopy (UVFD; DermLite DL5 Wood mode 365 nm) exhibited blue–white autofluorescence of keratin flakes and milky-white fluorescence corresponding to a collagenous/myxoid stromal component (Figure 2). These findings were consistent with a locally aggressive tumour causing local neoangiogenesis with a myxoid, collagenous component.

**Figure 3:**
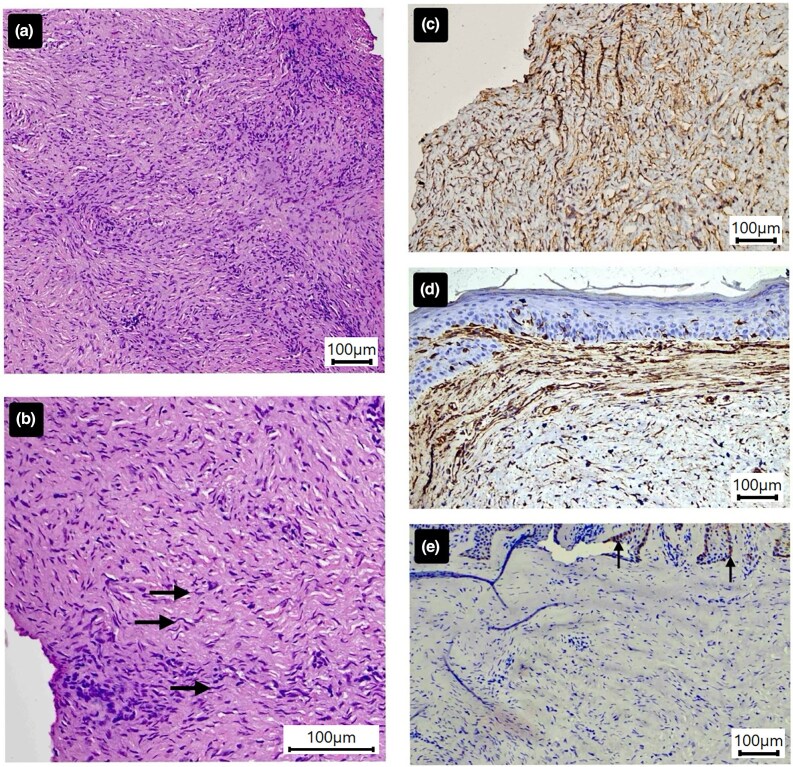
(a) Haematoxylin and eosin (H&E)-stained punch biopsy section demonstrating a diffuse monomorphic infiltrate of spindle cells embedded in a pale myxoid stroma with interlacing collagen bundles (×10 ocular lens coupled with a ×10 objective providing an effective magnification of ×100). (b) Higher magnification H&E-stained section (×10 ocular lens coupled with ×20 objective, providing an effective magnification of ×200), highlighting spindle-shaped cells with wavy nuclei, suggestive of neural origin (black arrows). (c) Immunohistochemical (IHC) staining for CD34 reveals diffuse positivity among spindle cells (×10 ocular lens coupled with a ×10 objective providing an effective magnification of ×100). (d) IHC-stained section for vimentin demonstrates strong cytoplasmic brown staining in all mesenchymal elements (×10 ocular lens coupled with a ×10 objective providing an effective magnification of ×100). (e) IHC staining for S100 shows no reactivity in tumour cells, with strong staining observed in basal melanocytes (black arrows; positive internal control) (×10 ocular lens coupled with a ×10 objective providing an effective magnification of ×100).

Histopathological examination of a punch biopsy, stained with haematoxylin and eosin (H&E), revealed a monomorphic proliferation of spindle-shaped cells with minimal atypia arranged in interlacing fascicles, infiltrating the dermis and obscuring the subcutis amidst a pale-staining stroma (Figure 3a, b). Although these features initially suggested a benign neurofibroma, the interpretation conflicted with clinical and dermoscopic findings, prompting further investigation.

**Figure 4:**
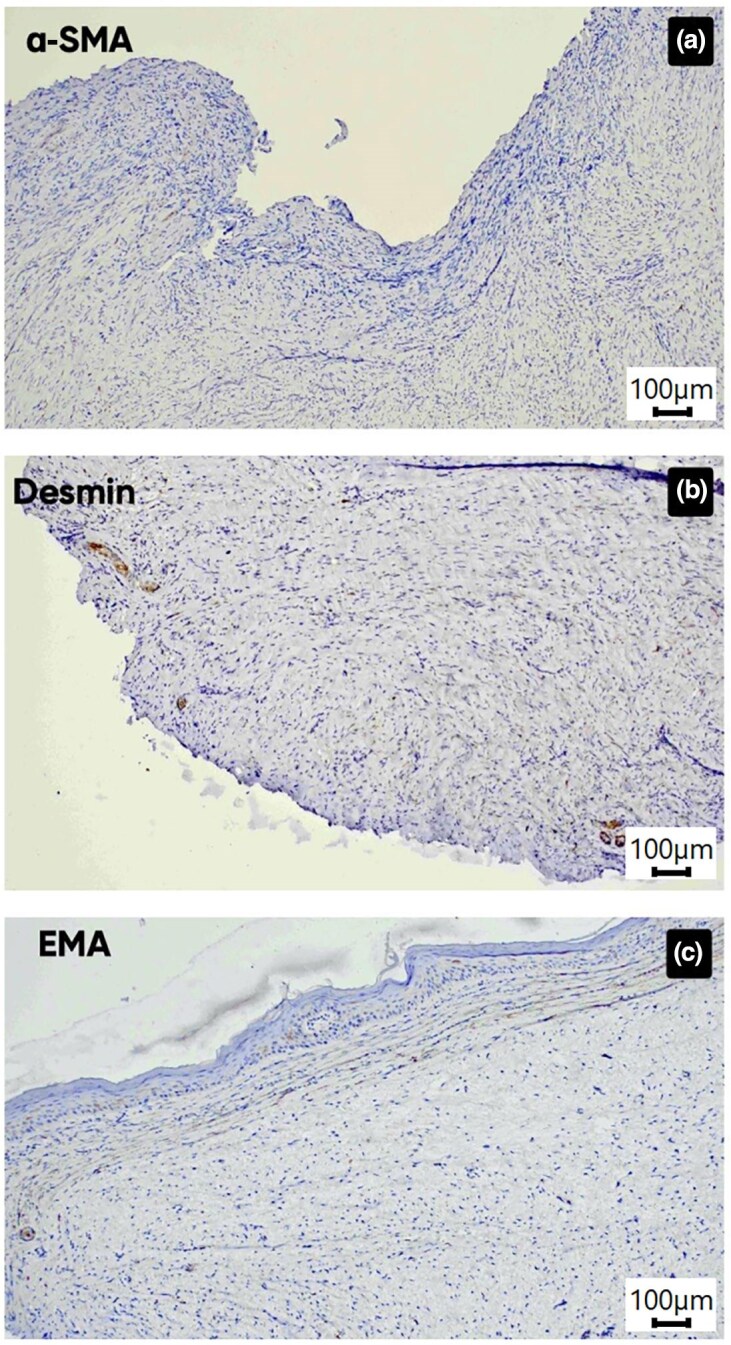
(a) Immunohistochemistry for α-smooth muscle actin (SMA) showing absence of immunoreactivity throughout the lesion (×10 ocular lens coupled with a ×10 objective providing an effective magnification of ×100). (b) Section stained for desmin demonstrating positivity restricted to larger vascular structures, likely representing vascular smooth muscle cells, with no staining in the neoplastic spindle cells (×10 ocular lens coupled with a ×10 objective providing an effective magnification of ×100). (c) Section stained for epithelial membrane antigen (EMA) showing no immunoreactivity within the spindle-cell population (×10 ocular lens coupled with a ×10 objective providing an effective magnification of ×100).

Immunohistochemistry (IHC) demonstrated strong diffuse CD34 and vimentin positivity within spindle cells and stroma, while S100 was limited to melanocytes (Figure 3c–e). Negative α-smooth muscle actin, desmin and epithelial membrane antigen ruled out myogenic and epithelial tumours, thereby helping confirm DFSP (Figure 4). The patient underwent wide local excision with 2–3-cm margins including fascia; frozen sections confirmed clear margins and recovery was uneventful.

## Discussion

DFSP encompasses multiple histological variants – myxoid, pigmented (Bednar tumour), myoid, granular cell, sclerosing, atrophic and fibrosarcomatous – many lacking the classic storiform (cartwheel) pattern and ‘honeycomb’ entrapment of subcutaneous fat, leading to frequent misdiagnosis as benign spindle-cell tumours. In particular, the myxoid variant, defined by pale myxoid stroma and collagen deposits, often mimics neurofibroma or myxoid liposarcoma on H&E sections, as reflected by our initial diagnostic uncertainty.^5^

The varied clinical presentation of DFSP necessitates consideration of multiple differential diagnoses. Table S1 (see Supporting Information) summarizes these entities, delineating key clinical, histopathological and IHC distinctions to guide accurate differentiation.

A genetic analysis of 53 patients with DFSP by Peng *et al*. reported frequent amplification of the *AKT1*, *SPHK1*, *COL1A1* and *PDGFB* oncogenes, particularly on chromosomes 17q25 and 22q13, with the *COL1A1–PDGFB* [t(17;22)] fusion present in 74% of cases.^6^ Notably, this fusion gene is also found in certain cases of acute lymphoblastic leukaemia and prostate carcinoma. Additional alterations included *SLC2A5–BTBD7* [t(1;14)] and mutations in *MUC4* and *MUC6*, *KMT2C* and *BRCA1*.^6^ Han *et al*. further reported *COL1A1–PDGFB* in 94.1% of cases, with diagnostic confirmation achievable by multiplex reverse transcriptase polymerase chain reaction for the t(17;22) translocation or fluorescence *in situ* hybridization for fusion gene detection.^7^

In this case, multimodal dermoscopy provided key diagnostic insights complementing histopathological ambiguity. Cross-polarized dermoscopy revealed thick arborizing vessels on a pinkish background with white structureless areas, suggestive of DFSP. Yellow light dermoscopy (580 nm) enhanced pigment networks and vascular channels as high-contrast dark structures, while UVFD highlighted keratin and collagen-rich myxoid stroma, guiding targeted biopsy.^8,9^ A review by Escobar *et al*. reported fine pigmented networks (78%), arborizing vessels (81%), structureless areas (44%), pink background (66%) and depigmented areas (50%) in DFSP.^10^ Although no single dermoscopic feature is pathognomonic, their combination improves diagnostic accuracy and biopsy site selection in equivocal cases.

Although multimodal dermoscopy serves as a quick bedside test for identifying DFSP, high-frequency ultrasound (HFUS) provides complementary insight by visualizing deeper architecture. DFSP typically demonstrates a heterogeneous hypoechoic subcutaneous mass with moderate peripheral vascularity on Doppler imaging.

Emerging technologies like the deep multimodal fusion network (DMFN) are further enhancing diagnostic precision. In a multicentre study involving 422 cases across 17 skin diseases, Zhu *et al*. demonstrated that this artificial intelligence model – which integrates clinical and HFUS images – achieved superior diagnostic accuracy (area under the curve 0.876) than general dermatologists.^11^

In binary classification tasks using HFUS (e.g. benign vs. malignant), the DMFN outperformed general practitioners and dermatologists but remained inferior to that of dermatologists specialized in HFUS. Using only close-up clinical images, it matched general dermatologists for benign lesions and surpassed them for malignant ones. However, when HFUS data were incorporated, HFUS-trained dermatologists performed better than the DMFN.

In multiclass classification tasks – which involved making specific diagnoses among 17 types of skin diseases – the DMFN exceeded the performance of general dermatologists using clinical images alone and achieved accuracy comparable to HFUS-specialized dermatologists.

While such advanced modalities represent significant progress, multimodal dermoscopy remains an accessible, noninvasive first-line diagnostic tool – particularly in resource-limited settings lacking advanced imaging.

Surgical excision remains the mainstay of DFSP treatment. Criscito *et al*. identified male sex, older age and larger tumour size as adverse prognostic factors, while treatment modality – although influenced by demographic factors – was not independently associated with altered ­survival.^12^

Surgical options include wide local excision (WLE) and Mohs micrographic surgery (MMS), with evidence favouring MMS. In a 10-year UK retrospective study of 547 cases, Durack *et al*. reported that WLE was performed in 75% of primary cases and MMS in 20%.^13^ All six local recurrences occurred after WLE, with none after MMS. First-attempt clearance was higher with MMS (87% vs. 81%), although the shorter follow-up (in the MMS group) and retrospective design limit definitive conclusions. WLE remains common, guided by preference, expertise and cost-effectiveness.

In another study, Fields *et al*. analysed 244 patients with DFSP (197 primary, 47 recurrent) and found that tumour depth was the strongest predictor of recurrence in primary DFSP (hazard ratio 3.14, *P* = 0.022), while margin status (R0 vs. R1) was decisive in recurrent cases (hazard ratio 22.43, *P* < 0.0001).^14^ Five-year disease-free survival was 92% for primary tumours and 87% for recurrent tumours. Systemic therapy (imatinib) induced partial responses in five of six advanced cases but provided only temporary control (6–36 months), reaffirming complete surgical excision with negative margins as the curative standard. Current guidelines recommend clinical follow-up every 6–12 months, with imaging or rebiopsy of any suspicious areas, given that margin status and tumour depth are key predictors of recurrence.^15^

In atypical or myxoid DFSP variants with equivocal histology, multimodal dermoscopy aids biopsy site selection, while IHC profiling remains vital for confirming diagnosis and planning surgery. This case highlights the importance of incorporating multimodal dermoscopy into routine skin examinations. Future studies should aim to standardize combined dermoscopy–IHC protocols for early detection, subtype distinction and optimal management.

Multimodal dermoscopy (combining cross-polarized, yellow light and UVFD) provides better visualization of pigmentary networks, vascular architecture and collagenous/­myxoid stroma, thereby guiding targeted biopsies and improving diagnostic accuracy in atypical DFSP. Myxoid and atrophic variants frequently lack classic storiform histology, mimicking benign spindle-cell neoplasms; IHC confirmation with diffuse CD34 positivity and S100 negativity remains essential to distinguish DFSP from neurofibromas and other histological mimics.

## Supplementary Material

vzaf113_Supplementary_Data

## Data Availability

All data are available in the article.
