# Supplementary material for: Highlighting a histopathological dilemma: atypical dermatofibrosarcoma protuberans diagnosed with multimodal dermoscopy
Source: Skin Health Dis. 2026 Jan 28;6(2):171–6. doi: 10.1093/skinhd/vzaf113 (PMC13036734; doi:10.1093/skinhd/vzaf113)
Supplement: vzaf113_Supplementary_Data [file vzaf113_supplementary_data.zip › FURTHERREADING.docx]

**APPENDIX S1 (SUPPORTING INFO) –**

**FURTHER READING –**

1. AlQusayer M, AlQusayer M, Alkeraye S. Unusual presentation of dermatofibroma on the face: Case report. Clin Case Rep. 2019 Feb 19;7(4):672–4.
2. Reimann JDR, Fletcher CDM. Myxoid dermatofibrosarcoma protuberans: a rare variant analyzed in a series of 23 cases. Am J Surg Pathol. 2007 Sep;31(9):1371–7.
3. Camara MF, Pinheiro PMR, Jales RD, da Trindade Neto PB, Costa JB, de Sousa VLLR. Multiple Dermatofibromas: Dermoscopic Patterns. Indian J Dermatol. 2013;58(3):243.
4. Zaballos P, Puig S, Llambrich A, Malvehy J. Dermoscopy of Dermatofibromas: A Prospective Morphological Study of 412 Cases. Archives of Dermatology. 2008 Jan 1;144(1):75–83.
5. Jartarkar SR, Spoorthy B, Kareddy S. Solitary Neurofibroma over Lower Lip: A Rare Manifestation. J Cutan Aesthet Surg. 2022;15(2):189–92.
6. Messersmith L, Krauland K. Neurofibroma. In: StatPearls [Internet]. Treasure Island (FL): StatPearls Publishing; 2025 [cited 2025 July 22]. Available from: <http://www.ncbi.nlm.nih.gov/books/NBK539707/>
7. Zhou Arlene Y, Chin J, Strutin MD, Lomiguen CM. Unmasking dermatofibrosarcoma protuberans: Case report of an atypical presentation complicated by post-surgical excision. Int J Surg Case Rep. 2020;69:101–4.
8. Kolb L, Schmieder GJ. Atypical Fibroxanthoma. In: StatPearls [Internet]. Treasure Island (FL): StatPearls Publishing; 2025 [cited 2025 July 25]. Available from: <http://www.ncbi.nlm.nih.gov/books/NBK459342/>
9. UpToDate [Internet]. [cited 2025 July 25]. Available from: <https://www.uptodate.com/contents/atypical-fibroxanthoma#H2664264209>
10. Yu M, Liang S, Wang W. Atypical fibroxanthoma in elderly people: A case report and literature review. Foot & Ankle Surgery: Techniques, Reports & Cases. 2023 Dec 1;3(4):100319.
11. Piras V, Ferreli C, Atzori L, Pinna G, Pilloni L. Atypical fibroxanthoma/pleomorphic dermal sarcoma of the scalp with aberrant expression of HMB-45: a pitfall in dermatopathology. Pathologica. 2020 June 1;112(2):105–9.
12. Hardisson D, Cuevas-Santos J, Contreras F. Solitary fibrous tumor of the skin. Journal of the American Academy of Dermatology. 2002 Feb 1;46(2, Supplement 1):S37–40.
13. Han SS, Park SK, Jang JW, Kim TL, Choi HS, Park HK, et al. Primary Cutaneous Solitary Fibrous Tumor on the Back. Ann Dermatol. 2020 Apr;32(2):155–8.
14. Tariq MU, Din NU, Abdul-Ghafar J, Park YK. The many faces of solitary fibrous tumor; diversity of histological features, differential diagnosis and role of molecular studies and surrogate markers in avoiding misdiagnosis and predicting the behavior. Diagnostic Pathology. 2021 Apr 20;16(1):32.
15. Chen LL, Jaimes N, Barker CA, Busam KJ, Marghoob AA. Desmoplastic Melanoma: A Review. J Am Acad Dermatol. 2013 May;68(5):825–33.
16. Busam KJ. Desmoplastic melanoma. Clinics in Dermatology. 2025 May 1;43(3):341–7.
17. Zaballos P, Del Pozo LJ, Argenziano G, Medina C, Lacarrubba F, Ferrer B, et al. Dermoscopy of cutaneous smooth muscle neoplasms: a morphological study of 136 cases. J Eur Acad Dermatol Venereol. 2019 Apr;33(4):693–9.
18. Kim NG, Kim JO, Park YJ, Kim JS, Lee YJ, Lee KS. Cutaneous Leiomyosarcoma of the Face. Arch Craniofac Surg. 2017 June;18(2):145–8.
19. Ciurea M, Georgescu C, Radu C, Georgescu C, Stoica L. Cutaneous leiomyosarcoma – Case report. J Med Life. 2014 June 15;7(2):270–3.
20. Soares Queirós C, Filipe P, Soares de Almeida L. Cutaneous leiomyosarcoma: a 20-year retrospective study and review of the literature. Anais Brasileiros de Dermatologia. 2021 May 1;96(3):278–83.
21. Wang H, Wang D, Jia L, Wang M, Zhang X, Shu P. Cutaneous malignant peripheral nerve sheath tumor – A case report and literature review. Interdisciplinary Neurosurgery. 2022 June 1;28:101492.
22. Hao X, Billings SD, Wu F, Stultz TW, Procop GW, Mirkin G, et al. Dermatofibrosarcoma Protuberans: Update on the Diagnosis and Treatment. Journal of Clinical Medicine. 2020 June 5;9(6):1752.
23. Shi BJ, Jiang X, Xiao YJ, Wang SP, Hao J, Diao QC. Intradermal spindle cell/pleomorphic lipoma: Case report and review of the literature. Indian J Dermatol Venereol Leprol. 2017 Nov 1;83:692.
24. Kwon HM, Yim JE, Kim HR, Shin DH, Choi JS, Bae YK, et al. Intradermal Low-Fat Spindle Cell Lipoma: A Case Report. Annals of Dermatology. 2023 May 3;35(Suppl 1):S10.
25. Lee RKL, Griffith JF, Ng AWH, Lai FMM. Ultrasound Appearances of Dermatofibrosarcoma Protuberans. Journal of Medical Ultrasound. 2013 Mar 1;21(1):21–8.
26. Lim SX, Ramaiya A, Levell NJ, Venables ZC. Review of dermatofibrosarcoma protuberans. Clinical and Experimental Dermatology. 2023 Apr 1;48(4):297–302.
27. Bernard J, Poulalhon N, Argenziano G, Debarbieux S, Dalle S, Thomas L. Dermoscopy of dermatofibrosarcoma protuberans: a study of 15 cases. British Journal of Dermatology. 2013 July 1;169(1):85–90.
28. Saiag P, Lebbe C, Brochez L, Emile JF, Forsea AM, Harwood C, et al. Diagnosis and treatment of dermatofibrosarcoma protuberans. European interdisciplinary guideline – update 2024. European Journal of Cancer. 2025 Mar 11;218:115265.
