# Supplementary figures and images for: Highlighting a histopathological dilemma: atypical dermatofibrosarcoma protuberans diagnosed with multimodal dermoscopy
Source: Skin Health Dis. 2026 Jan 28;6(2):171–6. doi: 10.1093/skinhd/vzaf113 (PMC13036734; doi:10.1093/skinhd/vzaf113)

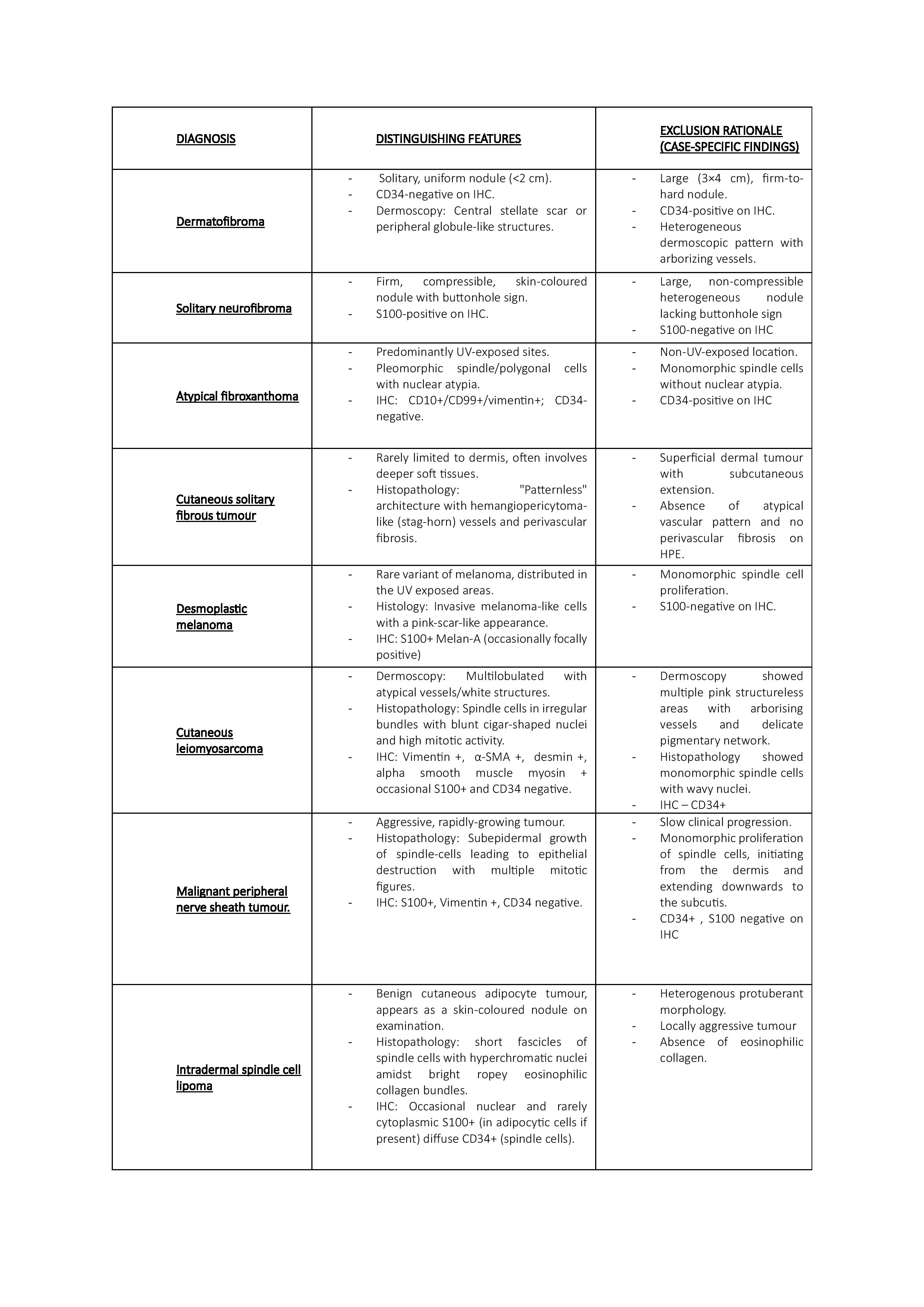

Supplement: vzaf113_Supplementary_Data [file vzaf113_supplementary_data.zip › Supplimentary information table 1.jpg]
